# Use of a Novel Clinical Decision Support Tool for Pharmacist-Led Antimicrobial Stewardship in Patients with Normal Procalcitonin

**DOI:** 10.3390/pharmacy9030136

**Published:** 2021-08-06

**Authors:** Andrew B. Watkins, Trevor C. Van Schooneveld, Craig G. Reha, Jayme Anderson, Kelley McGinnis, Scott J. Bergman

**Affiliations:** 1 Department of Pharmaceutical and Nutritional Care, Nebraska Medicine, Omaha, NE 68198, USA; creha@nebraskamed.com (C.G.R.); jaanderson@nebraskamed.com (J.A.); kmcginnis@nebraskamed.com (K.M.); scbergman@nebraskamed.com (S.J.B.); 2 Department of Internal Medicine, Division of Infectious Diseases, University of Nebraska Medical Center, Omaha, NE 68198, USA; tvanscho@unmc.edu

**Keywords:** antimicrobial stewardship, procalcitonin, pharmacy, clinical decision support

## Abstract

In 2018, a clinical decision support (CDS) tool was implemented as part of a “daily checklist” for frontline pharmacists to review patients on antibiotics with procalcitonin (PCT) <0.25 mcg/L. This study used a retrospective cohort design to assess change in antibiotic use from pharmacist interventions after this PCT alert in patients on antibiotics for lower respiratory tract infections (LRTI). The secondary outcome was antibiotic days of therapy (DOT), with a subgroup analysis examining antibiotic use and the length of stay (LOS) in patients with a pharmacist intervention. From 1/2019 to 11/2019, there were 165 alerts in 116 unique patients on antibiotics for LRTI. Pharmacists attempted interventions after 34 (20.6%) of these alerts, with narrowing spectrum or converting to oral being the most common interventions. Pharmacist interventions prevented 125 DOT in the hospital. Vancomycin was the most commonly discontinued antibiotic with an 85.3% use reduction in patients with interventions compared to a 27.4% discontinuation in patients without documented intervention (*p* = 0.0156). The LOS was similar in both groups (median 6.4 days vs. 7 days, *p* = 0.81). In conclusion, interventions driven by a CDS tool for pharmacist-driven antimicrobial stewardship in patients with a normal PCT resulted in fewer DOT and significantly higher rates of vancomycin discontinuation.

## 1. Introduction

Up to half of antimicrobial use in the inpatient setting is unneeded or inappropriate, and it is likely that treatment durations are excessive [1,2]. The Centers for Disease Control and Prevention has recommended antimicrobial stewardship as a method to limit inappropriate antibiotic use and control the spread of antibiotic resistant bacteria. Starting in 2017, The Joint Commission has mandated that acute care facilities implement Antimicrobial Stewardship Programs as a means to combat this public health emergency. The injudicious use of antibiotics and its propensity to cause antimicrobial resistance and patient harm has contributed to the growing need for tools, biomarkers, and innovative strategies to help improve antimicrobial prescribing.

Procalcitonin (PCT) is a precursor of calcitonin, a hormone normally produced in neuroendocrine C-cells of the thyroid and K-cells of the lung. Under normal conditions, PCT serum concentrations are <0.01 mcg/L; however, in the presence of systemic inflammation, and particularly bacterial infection, PCT is constitutively released from all parenchymal tissues and cell types in the body, resulting in a several-fold increase in serum concentrations [3]. The rise in PCT serum concentrations begins at 4 h after systemic infection onset with a peak at 8–24 h, making it a promising biomarker for systemic infection, particularly when compared to C-reactive protein’s much slower rise and peak around 36 h [4]. In addition to this prompt rise, PCT also has a rapid decline in serum concentrations with control of infection (assuming adequate renal function) due to its relatively short half-life of 24 h. The other characteristics making PCT a useful biomarker are its specificity for bacterial infection, correlation with disease severity, and lack of impairment by neutropenia or immunosuppressive medications (i.e., corticosteroids) [3,4,5].

The literature has supported the utility of PCT as a biomarker, particularly in sepsis and lower respiratory tract infections (LRTI) [6,7,8,9,10,11,12]. When these infections are suspected, the use of PCT to guide the duration of antibiotics leads to a decrease in antimicrobial utilization (19–38% reduction) and the duration of therapy (1.3-day decrease) without a worsening of clinical outcomes [13,14,15]. In fact, meta-analyses have found that PCT-guided antibiotic therapy is associated with significantly decreased mortality in both sepsis and acute respiratory infections, potentially due to the consideration of alternate diagnoses earlier in hospital admissions [16,17].

Our institution was an early adopter of PCT as a biomarker, with the Antimicrobial Stewardship Program (ASP) incorporating a review of patients on antibiotics with normal procalcitonin (<0.25 mcg/L) into its workflow since 2012. To empower clinical pharmacists, a clinical decision support (CDS) tool in the form of a pharmacist “Daily Checklist” alert was implemented in the electronic health record (EHR) in 2018. ASP subsequently gave primary ownership of this antimicrobial stewardship activity to clinical pharmacists.

While data exist highlighting the utility of pharmacist-driven procalcitonin protocols, the impact of a clinical-decision support tool such as the pharmacist Daily Checklist embedded in an electronic health record has not been as well documented [18,19,20]. This project aims to validate the effectiveness of this clinical decision support tool following the conversion from ASP-driven interventions to clinical pharmacist-led stewardship.

## 2. Materials and Methods

This retrospective cohort study at a 2-hospital, 809-bed academic medical center in the United States evaluated the impact of a CDS tool for pharmacist-driven, PCT-based antimicrobial stewardship in patients with LRTIs. Patients were included if they were receiving antibiotics for a suspected or confirmed LRTI and had a PCT Daily Checklist alert from January 2019 to November 2019.

The Daily Checklist is part of an overarching CDS tool created within our EHR (Epic OneChart) that utilizes rule-based logic to alert frontline clinical pharmacists when patients meet specific criteria. This tool, the “Pharmacist Checklist”, serves as a review activity to display alerts related to a patient’s clinical, educational, and discharge needs identified automatically by the EHR. There are numerous different alerts within this Daily Checklist that are used to bring attention to key issues or patient therapy opportunities at our medical center. Some examples of these rules include oral therapy conversion opportunities, warfarin therapy administered without monitoring orders being placed, sustained hyperglycemia alerts, and opioid reversal agents being administered. These alerts are set to present every day, including weekends, with the expectation of pharmacist review and/or intervention as needed for each alert by the end of their shift. Once addressed by the pharmacist, the alerts deactivate until an additional view is necessary, depending on the outcome of the initial review.

**Figure 1:**
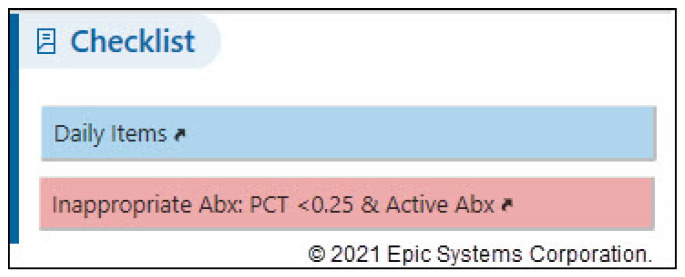
PCT daily checklist alert.

**Figure 2:**
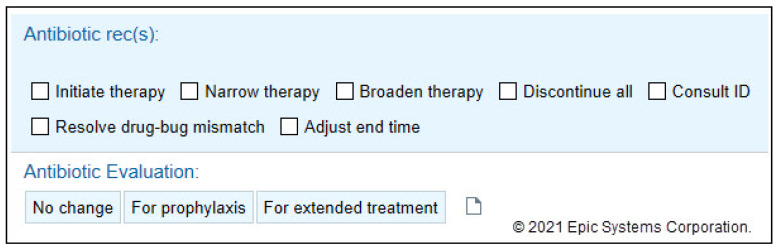
Pharmacist quick action documentation.

**Figure 3:**
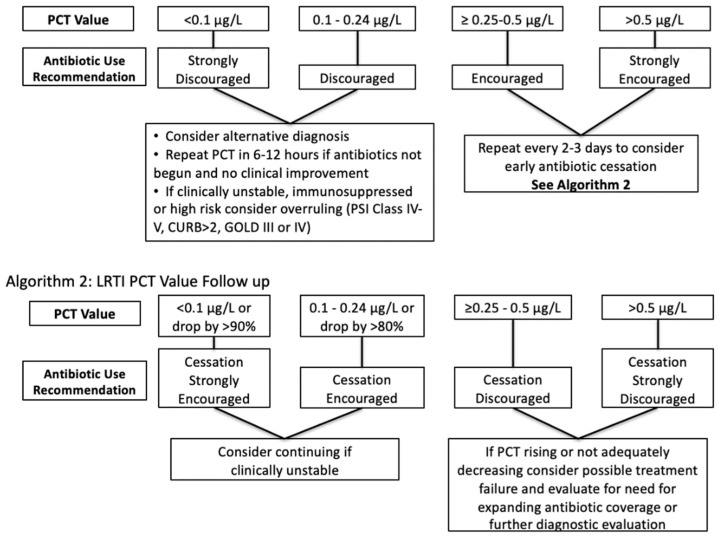
Institutional PCT algorithms for LRTIs.

The PCT-focused Daily Checklist alert presents on any patient with a PCT result <0.25 mcg/L in the preceding 3 days PLUS an active order for an antibiotic PLUS no abnormal (i.e., positive) culture results (Figure 1). Once this alert fires, patients should be assessed by team-based clinical pharmacists, who then either directly contact providers or discuss antibiotic therapy during patient care rounds. Pharmacists utilize a list of “Quick Action” options (narrow therapy, discontinue all antibiotics, adjust end time, etc.) to efficiently document any interventions. Another option includes “no change to current therapy”, which allows pharmacists to denote that the alert has been addressed/reviewed but no changes were made (Figure 2). Once this alert was created, ASP leaders provided a live educational presentation on the use of PCT to frontline clinical pharmacists. This was accompanied by the sharing of educational slides and institutional guidance and algorithms on the use of PCT (Figure 3).

A report of all patient charts with a PCT alert in the study period were obtained and narrowed to only those patients who also had a respiratory-related diagnosis (e.g., pneumonia, chronic obstructive pulmonary disease, respiratory failure, shortness of breath, cough, hypoxia, etc.) upon discharge. This was performed to focus on only those who potentially received antibiotics for LRTI, which is where PCT has the most utility for determining the need for antibiotics. An in-depth chart review was then performed on this narrowed list to ascertain the final dataset of patients receiving antibiotics specifically for suspected or confirmed LRTI. Repeat patients were included if additional alert(s) fired, as each alert represented an opportunity for pharmacist intervention.

The primary outcome was change in antibiotic use within 24 h of a pharmacist intervention. Antibiotic changes were classified based on the spectra of activity (narrowing, broadening), duration of therapy (shortening or extending course, discontinuation of all antibiotics), oral conversion, or dose optimization. The secondary outcome was antibiotic days of therapy (DOT). DOT were calculated as the sum of the calendar days each antibiotic was administered, and these were broken into categories of “DOT prevented”, “DOT optimized”, and “DOT not optimized”. DOT prevented was calculated as the difference between antibiotic DOT administered and the duration of the original antibiotic order. DOT optimized was defined as the DOT administered after an intervention that narrowed the spectrum, converted therapy to oral, or optimized antimicrobial doses. “DOT not optimized” was calculated as the DOT after an alert was marked as “no change” (i.e., no intervention was made on an eligible alert); these were not counted if there was an ID consult, positive respiratory culture occurring after an alert, or upward trend in PCT that would potentially preclude an intervention being made. A subgroup analysis was performed to compare antibiotic use and LOS in patients with a pharmacist intervention vs. those with a PCT alert but no documented pharmacist intervention.

Descriptive statistics were used to characterize antibiotic de-escalation and DOT, while comparisons between intervention groups were compared using chi-squared tests. This study was deemed exempt by the University of Nebraska Medical Center Institutional Review Board.

## 3. Results

**Figure 4:**
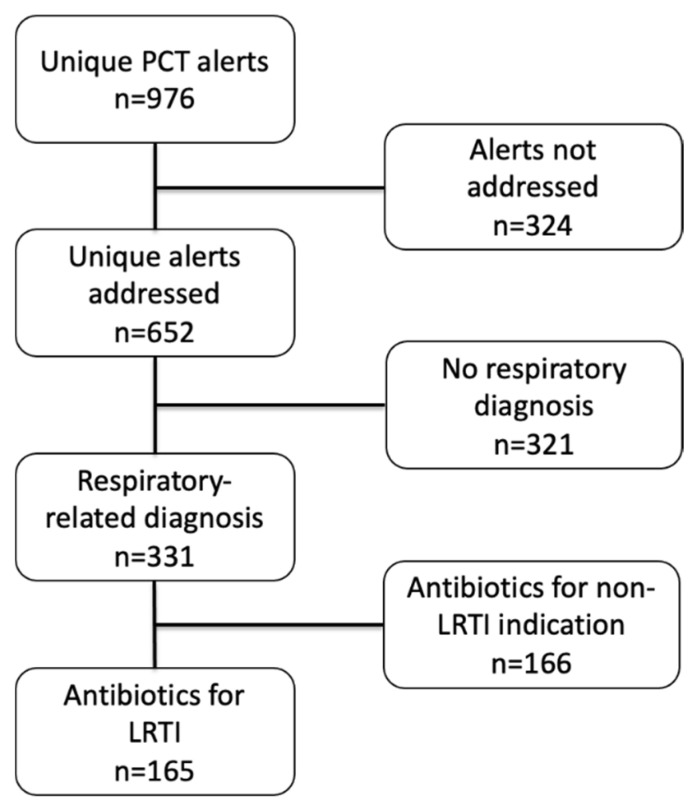
Inclusion flowchart.

In the 11-month study period, there were a total of 976 procalcitonin alerts, of which 652 (66.8%) were documented as addressed by pharmacists. Of these with documentation, 583 (89.4%) were marked as “No change to current therapy”. Of the 652 procalcitonin alerts, 331 occurred in patients with some sort of respiratory-related diagnosis. Another 166 of these alerts were excluded due to antibiotics being used for indications other than lower respiratory tract infections, leaving a final set of 165 PCT alerts in patients on antibiotics for respiratory tract infections occurring in 116 unique patients over 119 unique admissions (Figure 4). The most common treatment diagnoses associated with exclusion were urinary tract infection (*n* = 61, 36.7%), bacteremia (*n* = 38, 22.9%), and skin/soft tissue infection (*n* = 21, 12.7%). Patient demographics can be found in Table 1.

A total of 53 interventions were made or attempted by pharmacists after 34 of the 165 included alerts (20.6% intervention rate), with the most common interventions being narrowing therapy and converting from intravenous to oral. Some alerts had multiple interventions (e.g., oral conversion and shortening of duration), explaining the higher number of interventions than alerts. A breakdown of interventions can be found in Table 2. Two patients had their antibiotic therapy re-escalated within 48 h of pharmacist intervention.

In the 34 patients with pharmacist interventions, a total of 125 DOT were prevented and 56 DOT were optimized (i.e., narrower therapy, oral conversion, dose optimization). The alerts that were documented as “no change” by pharmacists (i.e., no change was made after an eligible alert) were associated with 140 DOT that were potentially preventable upon review.

In the subgroup analysis, the patients with a pharmacist intervention had significant reductions in vancomycin use (85.3% vs. 27.4% discontinuation), while having a similar LOS (6.4 vs. 7.0 days) compared to the patients without a pharmacist intervention (Table 3). There were no significant differences in the use of any other antibiotics (ceftriaxone, ceftazidime, cefepime, piperacillin/tazobactam, levofloxacin, azithromycin, doxycycline, amoxicillin/clavulanate, metronidazole, sulfamethoxazole/trimethoprim) between these two groups.

## 4. Discussion

This study evaluated the utility of a novel pharmacist-directed CDS tool in patients with normal procalcitonin. The use of this tool resulted in 181 days of therapy prevented or optimized with a 21% rate of intervention. These interventions were able to be implemented safely, with only two instances of therapy escalation within 48 h of the intervention and no change in the LOS in the patients receiving interventions vs. those not. This alert was also associated with a significant decrease in vancomycin use in patients who had a pharmacist intervention vs. those who did not. Since this project’s completion, at least one other study has been published evaluating the use of a CDS tool for pharmacist-driven procalcitonin protocols. This study found a decrease in antibiotic DOT with no change in LOS, further highlighting the utility of leveraging CDS for pharmacist-driven, procalcitonin-based antimicrobial stewardship [21].

While this tool has the potential to aid in antimicrobial stewardship, opportunities for improvement exist. With a higher intervention rate, there were more days of therapy administered after PCT alerts that could have potentially been prevented with a pharmacist’s intervention. One explanation for this small intervention rate could be alert fatigue, especially given the fact that over half of the alerts in this study occurred in patients not on antibiotics for lower respiratory tract infections. Refining the logic for this clinical decision support tool to exclude patients on antibiotics for urinary tract and skin/soft tissue infections, which have little supporting evidence for the use of PCT, could result in a more targeted and relevant alert that could increase the rate of intervention. Another intriguing finding was the relatively low rate of antibiotic discontinuations in our patients (7.5%). With the literature showing the value of using PCT to stop antibiotic therapy altogether, we would have hoped to see more outright discontinuation rather than just a narrowing of therapy or oral conversion. This perhaps highlights an area of opportunity for further pharmacist education or provider experience with the use of PCT and common practice not to start antibiotics in most patients with a normal result.

The limitations of this study include its retrospective, observational design and lack of a true comparator group. The reporting limitations within the EHR precluded the use of a well-matched control group to compare outcomes prior to the Daily Checklist implementation. The lack of thorough documentation and subjectivity of the PCT workflow were also limitations in this study. While pharmacists were able to select preset “Quick Actions” to document their interventions, there was typically no narrative documentation, requiring manual review of the medication administration record to confirm antibiotic changes around the time of the addressed alert. The PCT workflow is also driven by individual pharmacist judgment rather than a strict protocol, allowing for inconsistencies in interventions among pharmacists.

Despite these limitations, this study was able to show that interventions driven by a CDS tool for pharmacist-driven antimicrobial stewardship in patients with a normal PCT resulted in fewer DOT and significantly higher rates of vancomycin discontinuation. These findings highlight the utility of such a tool to broaden the reach of an antimicrobial stewardship program and empower frontline pharmacists to participate more actively in stewardship. Additional interventions could have potentially prevented 140 DOT, and we feel the refinement of this tool can lead to a more meaningful CDS, reduce alert fatigue, and likely increase intervention rates.

## Figures and Tables

**Table 1:** Patient characteristics at time of first alert.

Characteristics	*n* = 116
Mean age, years	62.9
Female, n (%)	57 (50.4)
Median LOS, days	6.84
Intensive care, n (%)	56 (48.3)

**Table 2:** Antibiotic changes after pharmacist intervention.

Type of Intervention	*n* (%)
Narrow therapy	18 (34)
IV-PO	10 (18.9)
Drug/dose optimization	8 (15.1)
Shorten duration	7 (13.2)
Discontinue all antibiotics	4 (7.5)
Prolong therapy	4 (7.5)
Broaden therapy	2 (3.8)
**Total**	**53**

**Table 3:** Subgroup analysis of patients with intervention vs. no intervention.

	Intervention	No Intervention	*p*-Value
Median LOS, days	6.4	7.0	0.81
Vancomycin discontinuation (rate)	85.3%	27.4%	**0.016**

## Data Availability

The data presented in this study are available on request from the corresponding author. The data are not publicly available due to confidentiality.
